# Supplementary material for: The interactome of intact mitochondria by cross-linking mass spectrometry provides evidence for coexisting respiratory supercomplexes
Source: Mol Cell Proteomics. 2017 Dec 8;17(2):216–32. doi: 10.1074/mcp.RA117.000470 (PMC5795388; doi:10.1074/mcp.RA117.000470)
Supplement: Supplemental Data [file supp_RA117.000470_133922_0_supp_23978_fzffwf.zip › spectra_annotation/mito_DR_spectra_annotation/264-1-3-1-3-1.pdf]

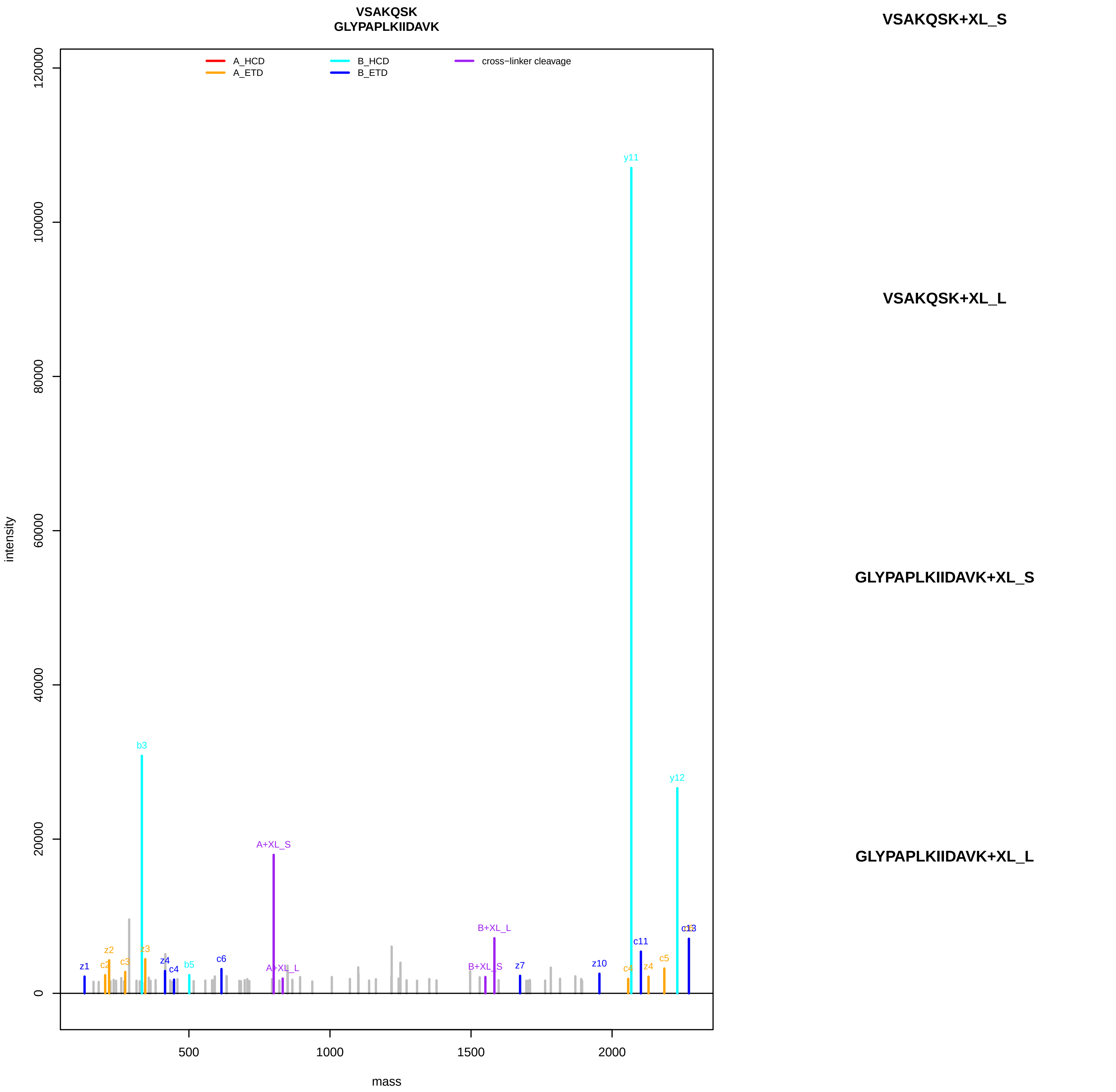
VSAKQSK
GLYPAPLKIIDAVK
A_HCD
A_ETD
B_HCD
B_ETD
cross−linker cleavage
intensity
mass
y11
b3
y12
A+XL_S
B+XL_L
A+XL_L
B+XL_S
c13
c11
c5
z4
c4
z10
z7
c6
b5
z4
c4
z3
z2
c3
c2
z1
VSAKQSK+XL_S
VSAKQSK+XL_L
GLYPAPLKIIDAVK+XL_S
GLYPAPLKIIDAVK+XL_L
